# Cross-Sectional Nakagami Images in Passive Stretches Reveal Damage of Injured Muscles

**DOI:** 10.1155/2016/6893712

**Published:** 2016-01-05

**Authors:** Shih-Ping Lin, Yi-Hsun Lin, Shih-Chen Fan, Bu-Miin Huang, Wei-Yin Lin, Shyh-Hau Wang, K. Kirk Shung, Fong-Chin Su, Chia-Ching Wu

**Affiliations:** ^1^Department of Biomedical Engineering, National Cheng Kung University, No. 1, University Road, Tainan 701, Taiwan; ^2^Department of Computer Science and Information Engineering, National Cheng Kung University, No. 1, University Road, Tainan 701, Taiwan; ^3^Department of Occupational Therapy, I-Shou University, No. 1, Sec. 1, Syuecheng Road, Dashu District, Kaohsiung, Taiwan; ^4^Department of Cell Biology and Anatomy, National Cheng Kung University, No. 1, University Road, Tainan 701, Taiwan; ^5^Institute of Basic Medical Sciences, National Cheng Kung University, No. 1, University Road, Tainan 701, Taiwan; ^6^Department of Biomedical Engineering, University of Southern California, Los Angeles, CA 90089, USA; ^7^Medical Device and Innovation Center, National Cheng Kung University, No. 1, University Road, Tainan 701, Taiwan

## Abstract

Muscle strain is still awanting a noninvasive quantitatively diagnosis tool. High frequency ultrasound (HFU) improves image resolution for monitoring changes of tissue structures, but the biomechanical factors may influence ultrasonography during injury detection. We aim to illustrate the ultrasonic parameters to present the histological damage of overstretched muscle with the consideration of biomechanical factors. Gastrocnemius muscles from mice were assembled and* ex vivo* passive stretching was performed before or after injury. After injury, the muscle significantly decreased mechanical strength. Ultrasonic images were obtained by HFU at different deformations to scan in cross and longitudinal orientations of muscle. The ultrasonography was quantified by echogenicity and Nakagami parameters (NP) for structural evaluation and correlated with histological results. The injured muscle at its original length exhibited decreased echogenicity and NP from HFU images. Cross-sectional ultrasonography revealed a loss of correlation between NP and passive muscle stretching that suggested a special scatterer pattern in the cross section of injured muscle. The independence of NP during passive stretching of injured muscle was confirmed by histological findings in ruptured collagen fibers, decreased muscle density, and increased intermuscular fiber space. Thus, HFU analysis of NP in cross section represents muscle injury that may benefit the clinical diagnosis.

## 1. Introduction

Muscle strains are a result of damage accrued during overstretching of muscles and are the most common injuries caused by passive or active forcible stretching [1]. Muscle injury is diagnosed by physical palpation to identify tenderness and swelling over the injury site and through measures of muscle strength. Identification and classification of different injury levels are difficult to achieve through regular physical examination. Methods published in 1969 [2] are the basis for the current classification of strain injury that grades severity by symptoms after injury. However, knowledge of the strain location, size, and injured muscle mechanical properties could be used to create more efficient therapies that achieve better prognosis.

Muscle tissue is a viscoelastic material that contains both elastic and viscosity behaviors [3]. When contractile tissue is subjected to mechanical loading, the resulting mechanical properties reflect the level of biomechanical function in the tissue [4]. Various components found within skeletal muscle contribute to mechanical properties under active and passive tension. Skeletal muscle tissues are composed of a network of primarily muscle fibers that enable contraction and collagen and elastin for cell embedding within the extracellular matrix (ECM) [5]. The ECM in muscle tissue is responsible for an increment stiffness modulus under passive tension [6]. The epimysium encloses the muscle tissue and the perimysium surrounds individual muscle fascicles, both of which consist primarily of type I and type III collagen [6–8]. A layer of ECM, the endomysium, surrounds the individual muscle fiber and is chiefly composed of type III collagen. All of these components contribute to mechanical force, which decreases in injured muscles exposed to passive stretch immediately after a strain injury. Mechanical properties of injured muscles also likely change as healing progresses [9]. However, to the best of our knowledge, no studies have investigated the damages and rearrangements of these microstructures with correlating to the biomechanical properties of an overstretching injured muscle.

Medical imaging systems, such as magnetic resonance imaging (MRI) and ultrasound imaging, are commonly used to define the location, extent, and size of strain injury [10–12]. Ultrasound is a well-developed medical application with advantages that include low costs, wide availability, portability, and noninvasive imaging modalities. Ultrasound can be used to immediately visualize structural details by converting acoustic impedance of soft tissue into an ultrasonographic image [13]. High frequency ultrasound (HFU), defined as ultrasound frequencies above 15 MHz, provides better image resolution for diagnosis and laboratory experiments [14]. HFU is currently used for imaging applications in ophthalmology, dermatology, and small animal studies [15]. Echogenicity, ultrasonic backscattered signals, can correspond with the microstructure of biological tissues, such as shape, size, concentration, density, and other elastic properties. Echogenicity increases with increasing density and with decreasing homogeneity [16]. Our recent findings suggest that echogenicity can also be used as an indicator for tissue inflammation during fracture healing [17]. Numerous studies have examined backscattered signals analyzed by Nakagami distribution analysis in ultrasonic pulse-echo images; these studies have found that the signals reflect the structure of the tissue and can predict tissue composition and complexity [18, 19]. In another study, HFU was used to quantitatively assess the orientation and distribution of carbon fibers by integrated backscatter (IB) and Nakagami statistical parameters [20]. Nakagami imaging also can combine with a strain-compounding technique to identify the elasticity characteristics of breast tumors [21]. By combining with the frequency and temporal compounding techniques, Nakagami imaging can be used to monitor the radiofrequency ablation in liver fibrosis [22]. The orientation of the muscle fascicle was determined using 2D ultrasound with a 3D position tracker system to find the 3D fascicle orientation in normal muscles [23]. However, ultrasonic characteristics of injured muscles were not analyzed.

Here, we aimed to establish a noninvasive method of detecting muscle tissue damage by high frequency ultrasound and to understand how quantifiable acoustic parameters change as a result of* ex vivo* muscle injury. We hypothesize that echogenicity and Nakagami images will be reflective of loading situations in muscles. The* ex vivo* experimental approaches used here allow us to directly measure muscle response to mechanical strain and to eliminate the noise of surrounding tissue in ultrasonic images. Understanding skeletal muscle mechanical behavior under different loading conditions using ultrasonography could improve the current classification system, provide better guidance for accurate diagnosis, and allow for more efficient treatments to be administered.

## 2. Materials and Methods 

### 2.1. *Ex Vivo* Models for Muscle Injury

**Figure 1:**
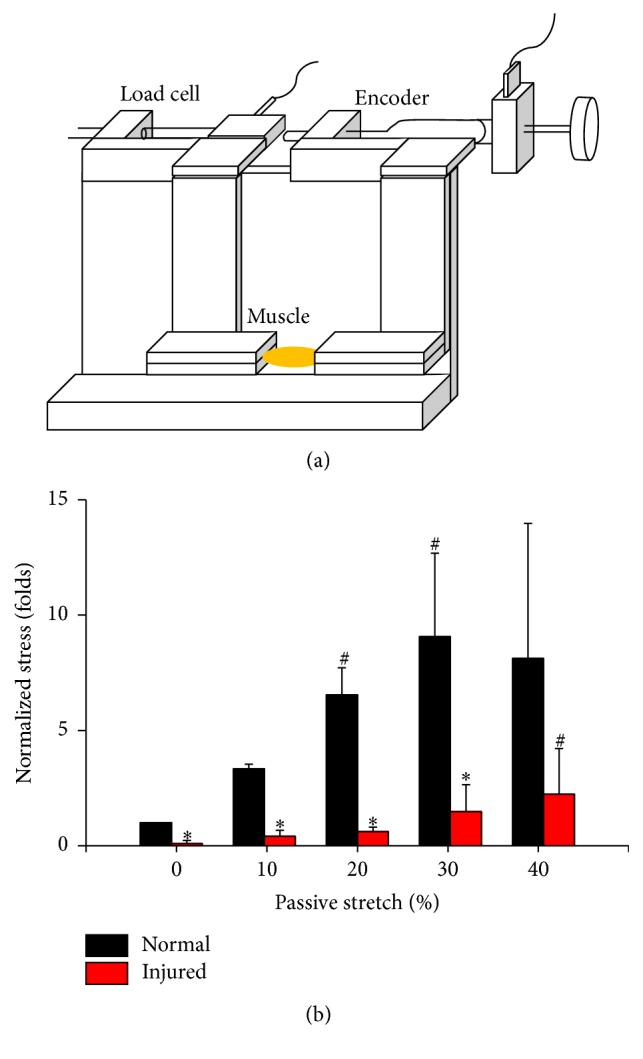
The uniaxial passive stretching system was developed to measure the mechanical properties of* ex vivo* gastrocnemius muscles (a). The muscle was isolated from mice. One side was clamped on the load cell and the other side was linked with the encoder to record displacement during different deformations (strains). In normal muscle, the increase of muscle length during passive stretching increased the normalized stresses (b). The overstretch injury significantly decreased the muscle strength (stress) at different deformations in 0, 10, 20, 30, and 40% passive stretches. #: significant difference (*P* < 0.05) from the original length in the same muscle. *∗*: significant difference (*P* < 0.05) from the normal muscle in the same strain.

The injury model for investigating biomechanical and ultrasonic properties in overstretched muscles was created in* ex vivo* gastrocnemius muscles isolated from 8-week-old female B6 (C57BL/6N) mice. Animals were supplied by the National Cheng Kung University (NCKU) animal center. The experimental procedures used for this study were reviewed and approved by the Institutional Animal Care and Use Committee (IACUC) at NCKU. Briefly, the mice were sacrificed by carbon dioxide (CO_2_) asphyxiation. Before isolating the gastrocnemius muscle, muscle length was measured by a caliper while placing the knee in 90° flexion and the ankle in 90° plantar flexion. The full length of the gastrocnemius muscle was then removed from both right and left hind limbs by cutting at the insertion bone. To eliminate the mechanical contribution of surrounding ECM, the epimysium was mechanically removed to measure the response of the gastrocnemius muscle to tensile force. Throughout the isolation and mechanical testing procedures, the isolated muscles were kept hydrated in phosphate-buffered saline (PBS) solution, and the experiments were finished within 2 hours. The isolated muscle was secured in custom clamps at two bony ends and assembled on a custom uniaxial stretching testing system developed in our laboratory (Figure 1). To create an overstretching injury, the muscle was deformed until reaching 140% of the original length and held for 5 minutes. This strain was selected to fall outside of normal maximal physiologic strain, but still below the failure point, and caused microdamage in the muscle tissue [24]. Immediately after the overstretch injury, the muscle tissues were released to original length and the relaxation test was performed in different deformations.

### 2.2. Biomechanical Properties of Normal and Injured Muscles

Passive stretch was performed by moving the distance between two clamps using the micrometer caliper with the minimized step-length of 10 *μ*m. The resulting displacement was recorded by an encoder with a resolution of 360CPR (HS302-360P-6A, Honest sensor, Taiwan). Mechanical loading was measured via a 10-lb load cell (Cooper Industries, USA) that was attached to one end of the micrometer caliper. The force during mechanical testing was continuously recorded as voltage by an analog-to-digital DAQ system (i555 starter system, InstruNet Inc.) with a sampling frequency of 200 Hz. The isolated muscle was preloaded to the original length to avoid slack during mechanical testing. The load cell continuously recorded tensile force for the phenomenon of stress relaxation with increments of 10% deformation. The mean tensile force was measured between three and seven minutes after deformation, when the muscle achieved static state. The mechanical forces of deformed muscles were recorded and converted into N (newton) units. Cross-sectional images were obtained by HFU. The cross-sectional areas of the muscles were quantified and mechanical strains were normalized to the muscle area for presenting stress-strain curves under different deformations.

### 2.3. High Frequency Ultrasound

The HFU system comprised a 30 MHz single-element ultrasound transducer (NIH Ultrasonic Transducer Resource Center, USC, LA, USA) for the generation and reception of ultrasound waves. Echo radio frequency signals were amplified by a low-noise amplifier (Model LN1000A, Amplifier Research, PA, USA), filtered with a bandpass filter (Model BIF-30, Mini-Circuits, NY, USA). An 8-bit analog-to-digital (A/D) converter (PXI 5152, National Instruments, TX, USA) was used to receive ultrasound waves and convert to digital data acquisition at a 500 MHz sampling frequency. The transducer was also able to flexibly move in two other directions controlled by two axes of stepping motors (CM1-C-17L30A, Cool Muscle, Osaka, Japan) and actuators (KR2602A, THK, Tokyo, Japan). All motor movements were controlled by a motor controller (DMC-1842, Galil Motion Control Inc., California, USA). The programs developed for data acquisition and motor control were implemented using LabVIEW software (National Instruments, TX, USA).

The scan directions were along or perpendicular to the long axis of muscle tissue and the image was displayed as either longitudinal or cross-sectional, respectively. To understand the ultrasonic properties during muscle deformation, three-dimensional ultrasonography images were acquired using serial scanning by moving the HFU transducer in both longitudinal and cross-sectional orientations. In cross-sectional images, the HFU acquired the B-mode image almost perpendicular to the orientation of gastrocnemius muscle fibers. Serial sections at 50 *μ*m scanning intervals per step were performed from proximal to distal of the muscle belly for collecting a total of 200 slides. The longitudinal images were obtained along the longitudinal axis of the gastrocnemius muscle and scanned from the medial to lateral side of the muscle with 50 *μ*m intervals per step. To avoid long-term scanning or damage of muscle tissue during scanning, all assembled muscles with clamps were kept in PBS and each muscle was only subjected to one direction of scanning. During serial scanning, the brightness/depth (B/D) mode was used to reduce adverse effects of beam diffraction on the image resolution [18].

The echogenicity and Nakagami distribution were quantified to illustrate the ultrasonic properties in normal and injured muscles. Four regions of interests (ROIs) were selected in cross-sectional and longitudinal images. In cross-sectional images, ROIs were selected at the center axis of the muscle, which corresponded to the force application. The other three areas were selected at the 3, 9, and 12 o'clock directions. The size of each ROI was 1 mm × 1 mm in original muscle length. When the muscle deformed during passive stretches, the ROI size gradually decreased in proportion to the new total area in a different deformed muscle. In the longitudinal sections, the ROIs were defined at the muscle belly (the thickest area), at two areas close to both muscle tendon junctions on the axis of force transmitted, and at a fourth area at the superficial part of the muscle belly. During passive stretches, the length of ROIs increased corresponding to the deformation (original length of ROI × (100% + deformation (%)). The width of ROIs decreased in correlation to the decreasing ratio of muscle thickness. ROIs in the ultrasound images were analyzed by averaging the gray-value and Nakagami parameter using MATLAB software. The Nakagami parameter, a shape parameter associated with the Nakagami distribution, was used to characterize tissue signals from normal and injured muscle. The Nakagami parameter was presented in pseudocolor from black to blue for 0 to 1, and from blue to red for 1 to 2.

### 2.4. Histological Assessment of Muscle Tissue

To demonstrate histological features of normal and injured muscles during passive stretch, muscle tissues were fixed while in different deformations using custom design clamps. Tissues were merged in 10% neutral buffered formalin (Leica, German) at 4°C for 24–48 hours. Tissue samples were then dehydrated by sequential immersion in gradient ethanol baths (50%, 75%, 85%, 90%, 95%, and 100%) [17]. The dehydrated tissue was cleaned by xylene twice for 30 minutes and then immersed in a wax cylinder for paraffin embedding. Paraffin blocks were cut at 5 *μ*m thickness in cross-sectional or longitudinal orientations corresponding to HFU scanning images. The relative sections collected by histological sections can be referred to the serial HFU images in* ex vivo* muscle tissue. Hematoxylin and eosin (H&E) staining was used to observe tissue morphology. The tissue sections were stained with hematoxylin (Leica, Germany) for 4 minutes and then eosin (Leica, Germany) for 20 seconds. After staining, tissue sections were dehydrated using ethanol and mounted with mounting xylene jell (Sigma).

Immunohistochemistry (IHC) staining was performed to identify protein expression of collagen types I and III in normal and injured muscles. Antigen retrieval was performed before IHC staining by placing the slides in Trypsin solution (0.05%) (GIBCO) at 37°C for 20 minutes. Nonspecific binding was blocked with 10% normal serum (FBS, Invitrogen) diluted in 0.02% Tris-buffered saline and tween 20 (TBSt) (Sigma) for 2 hours. Primary antibodies for collagen type I (1 : 200, Abcam, UK) and type III (1 : 200, Abcam) were diluted in TBS with 10% normal serum and incubated at 4°C overnight. After rinsing with 0.02% TBSt, the bound primary antibodies were tagged by secondary antibodies (1 : 500, Abcam) for 1 hour and then labeled using AB reagent (Vector, USA) for 10 min to couple them to DAB. Tissue sections were stained with hematoxylin counterstain and then mounted using mounting medium.

The density of muscle tissue was calculated according to the muscle fiber number in the ROI. The cross-sectional area of each muscle fiber was also calculated using MATLAB software. Briefly, the acquired color histological images were converted into grayscale to detect the edge of muscle fibers and displayed in binary mode by setting the threshold. The area beneath the boundary was filled and the muscle area was calculated in the intact muscle fibers without being cut by the image borders. The pixel number for zero scatterer using the Nakagami parameter was also quantified and averaged for the four ROIs in each deformation.

### 2.5. Statistical Analyses

The quantified results are represented as means ± standard deviation. The differences between normal and injured muscle under various deformations were analyzed by one-way ANOVA with the Bonferroni test for multiple comparisons. Differences were considered statistically significant at an alpha level of *P* < 0.05. Pearson correlation analysis was used to calculate the simple linear correlation between ultrasonic and biomechanics parameters. The relationships between ultrasonic and biomechanical parameters were determined by correlation coefficients (*r*). Correlation was considered weak with an* r* = 0–0.3, moderate with an* r* = 0.3–0.7, or strong with an* r* = 0.7–1.0. All statistical analyses were performed using OriginPro software (OriginLab, version 8.5).

## 3. Results 

### 3.1. Overstretch Injury Decreased Muscle Strength during Passive Stretch

The stress of muscle strength during passive stretch was measured from the recorded loading force and then divided to the cross-sectional area of the muscle belly. The strain during passive muscle stretch was given by the displacement of microcalipers. The stress-strain relationship between normal and overstretch injured muscles was compared from 0 to 40% of passive stretch (Figure 1(b)). To eliminate the individual variance of different muscles, the stresses of normal and injured muscles were normalized to original stress under original muscle length before the overstretch damage. The normal muscle tissues showed a nonlinear increment of stress-strain relationship before the passive stretch achieved 40% of muscle length. After the overstretch injury, the stress of injured tissues was lower than normal muscles at a passive stretch of 0, 10, 20, and 30% (Figure 1(b)).

### 3.2. Different Scanning Orientations Showed Various Ultrasonic and Biomechanical Characteristics in Injured Muscles

**Figure 2:**
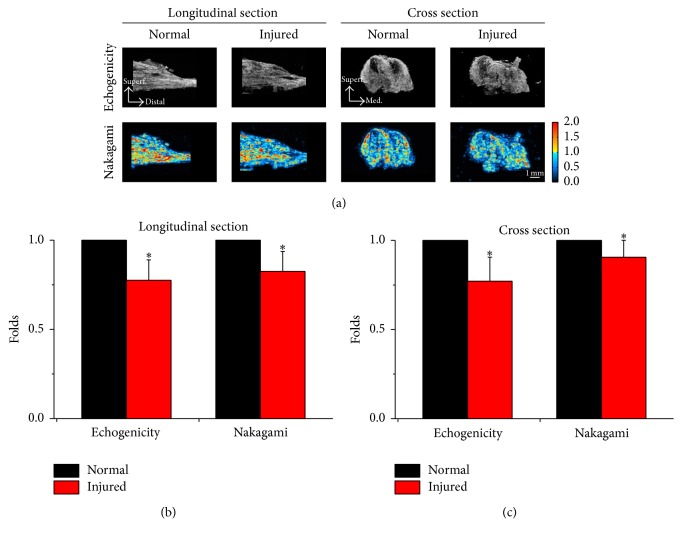
Ultrasonography was obtained by high frequency ultrasound (HFU) from longitudinal and cross sections of normal and injured muscles at the original length (a). The ultrasound images were presented as echogenicity (upper row) and Nakagami (lower row) images to indicate the tissue property. In longitudinal sections, both echogenicity and Nakagami were significantly decreased in the injured muscles (b). The injured muscle also showed decreased echogenicity and Nakagami parameters when acquiring the ultrasonic images in cross section (c). *∗*: significant difference (*P* < 0.05) from the normal muscle, bar = 1 mm.

To determine the best indication of muscle damage by ultrasonography, HFU images were acquired in both longitudinal and cross section at the original length of muscles before (normal) and after (injured) the overstretch injury (Figure 2(a)). In the longitudinal section, the ultrasonic parameters were displayed in echogenicity and Nakagami images in the midline of the gastrocnemius muscle to present the structures from the proximal attachment to the distal Achill's tendon. Ultrasonic images were also acquired on the middle portion of the muscle belly by cross-sectional scanning of normal and injured muscles. The injured muscle showed significant decreases of both echogenicity and Nakagami parameters regardless of the scanning orientation in longitudinal (Figure 2(b)) and cross section (Figure 2(c)).

**Figure 3:**
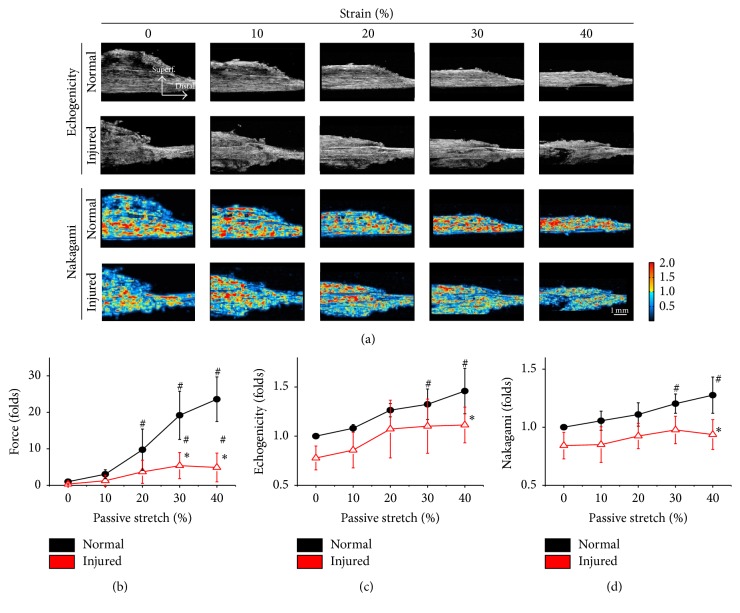
Passive stretches were sequentially applied with deformations of 0, 10, 20, 30, and 40% to the normal and injured muscles. After steady state was achieved in each deformation, the longitudinal HFU images were scanned and displayed in echogenicity (1st and 2nd rows) and Nakagami parameters (3rd and 4th rows) (a). The injured muscle showed decreased loading forces in 30 and 40% of passive stretch, as compared to the normal muscle in the same strain (*∗*), but it still showed increased force, compared to the original length of the injured muscle (#) (b). Increased echogenicity was observed in both normal and injured muscle deformations (c). The quantified Nakagami parameters also increased in passive stretched normal muscles (d). When comparing the longitudinal images between normal and injured muscles, the injured muscle only showed decreased echogenicity ((c), *∗*) and Nakagami parameters ((d), *∗*) at 40% deformation. #: significant difference (*P* < 0.05) from the original length in the same muscle. *∗*: significant difference (*P* < 0.05) from the normal muscle in the same strain.

Because overstretch caused muscle damage and resulted in decreasing stress during passive stretches, we further investigated whether change in ultrasonic parameters in different scanning orientations can reflect various muscle deformations. The muscle tissues were scanned in either longitudinal or cross section after static status was achieved at different strains with 0, 10, 20, 30, and 40% deformation in normal and injured muscles. In longitudinal section images, the ultrasonic characteristics of normal and injured muscles under different passive stretches are displayed as echogenicity (1st and 2nd rows) and Nakagami (3rd and 4th rows) images (Figure 3(a)). The mechanical loading (force) of injured muscles under 30 and 40% passive stretch was significantly decreased compared to normal tissue under the same deformation (Figure 3(b), *∗*). Differences in muscle length were maintained (Figure 3(b), #). In healthy muscle, the echogenicity was significantly raised during increasing strain in passive stretches (Figure 3(c)). The echogenicity of injured muscle showed the same tendency in longitudinal images and only expressed decreased echogenicity during 40% passive stretch (Figure 3(c)). Longitudinal Nakagami images also showed similar patterns in response to increased deformation in both normal and injured muscles (Figure 3(d)).

**Figure 4:**
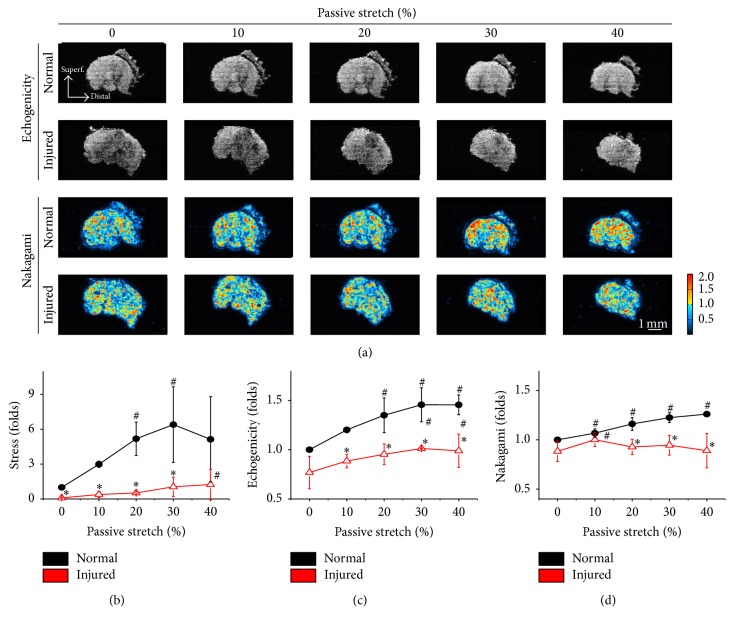
Passive stretch was sequentially applied to both normal and injured muscle as depicted in Figure 3, but HFU images were obtained by scanning the muscle in cross section (a). Normalized stresses during various passive stretches were derived from the loading force and muscle cross-sectional area measured from the HFU images (b). Significant differences were found between normal and injured muscles in each strain, except at 40% deformation. Significant decreases of echogenicity were observed in the cross-sectional images of injured muscles under different strains (c). Instead of showing increased patency of Nakagami parameters during increased strain, the cross-sectional Nakagami parameters were kept at the same level in the injured muscle (d). #: significant difference (*P* < 0.05) from the original length in the same muscle. *∗*: significant difference (*P* < 0.05) from the normal muscle in the same strain, bar = 1 mm.

Similar to the longitudinal images, cross-sectional HFU images were acquired in both normal and injured muscles subjected to strains from 0 to 40% passive stretch (Figure 4(a)). The HFU images of normal muscles showed decreased cross-sectional areas and increased echogenicity and Nakagami parameters during passive stretch. Mechanical forces during passive stretches were divided by the cross-sectional area of the muscle belly to present an evenly distributed stress among various strains (Figure 4(b)). The normalized stresses in the injured muscles significantly decreased at each level of stretch testing, but not at the 40% strain (Figure 4(b)). In healthy muscle, the echogenicity was significantly raised as deformation increased (Figure 4(c)). Although the cross-sectional area still decreased in the injured muscle, significant differences in echogenicity were observed between normal and injured muscles at 10, 20, 30, and 40% of strain levels. When high passive strains (30 and 40% strains) were achieved, increased echogenicity was still observed in damaged tissue, compared to injured muscle at the original length (Figure 4(c), #). The Nakagami parameters of cross-sectional images from injured muscles were also significantly different (Figure 4(d)). Interestingly, the Nakagami parameter in injured muscles did not reveal the increment tendency when responding to deformation, as demonstrated in normal muscles.

### 3.3. Cross-Sectional Ultrasonic Images Identify Muscle Injury Independent of Mechanical Properties

**Figure 5:**
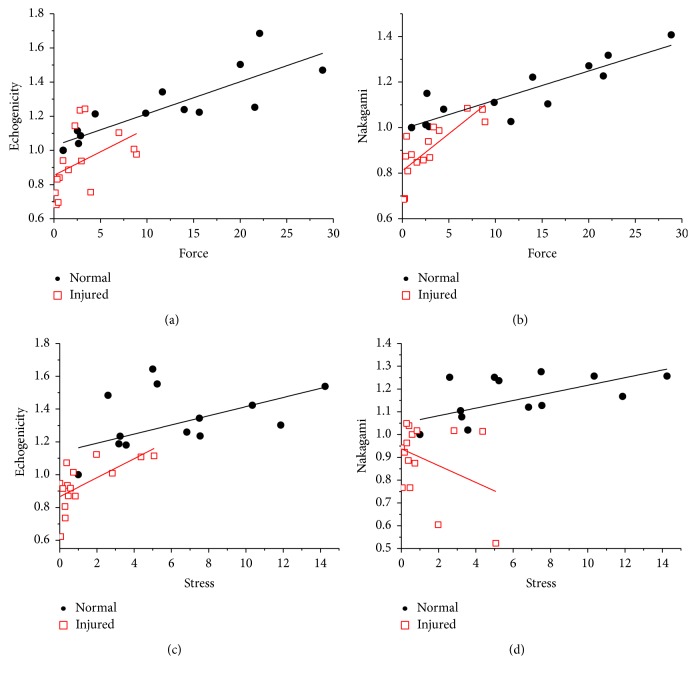
Regression analysis confirmed that mechanical forces were highly correlated to echogenicity (a) and Nakagami (b) parameters in longitudinal images for both normal (black solid squares) and injured (red open squares) muscles. The echogenicity also showed moderate correlation with mechanical stress in cross-sectional images (c). The injured muscle showed low correlation between cross-sectional Nakagami parameters and mechanical stresses (d).

To identify an ultrasonic parameter for distinguishing muscle injury regardless of mechanical factor influence, the correlation between ultrasonic and mechanical parameters in various strains is summarized for both normal and injured muscles. The relationship of mechanical stress to echogenicity and Nakagami parameters in normal and injured muscles was plotted using HFU images scanned in longitudinal (Figures 5(a) and 5(b)) and cross section (Figures 5(c) and 5(d)) orientations. The different increments of mechanical factors (forces or stresses) showed positive correlations with all ultrasonic parameters in both longitudinal and cross-sectional images in normal muscles (black solid circles). In longitudinal images, high positive correlations were observed in echogenicity (Figure 5(a), *r* = 0.86) and Nakagami (Figure 5(b), *r* = 0.9) for normal muscles. Even in the muscles that suffered overstretch injuries (red open squares), high positive correlations still existed between mechanical force and ultrasonic parameters (both echogenicity and Nakagami, *r* = 0.79) in longitudinal images. From the cross-sectional images, the echogenicity showed moderate positive correlations with stresses in both normal (*r* = 0.55) and injured (*r* = 0.65) tissues (Figure 5(c)). The Nakagami parameters of normal tissues were positively correlated to increased stresses with moderate association (*r* = 0.64), but this parameter was observed in negative correlation for injured muscle in cross-sectional images (*r* = −0.37) (Figure 5(d)). Our results confirmed that echogenicity reflects subject density, which may positively correlate to the loading situation of the muscles, especially in longitudinal images (Figure 5(a)). Nakagami images provide information related to structural integrity and distribution features in the scanning subject. The different correlation outcomes observed in Nakagami parameters between longitudinal (Figure 5(b)) and cross-sectional (Figure 5(d)) images indicated the importance of considering skeletal muscle structures and HFU scanning orientations.

### 3.4. Structural Behaviors in Injured Muscle Cause Independence of Cross-Sectional Nakagami Parameters under Deformation

**Figure 6:**
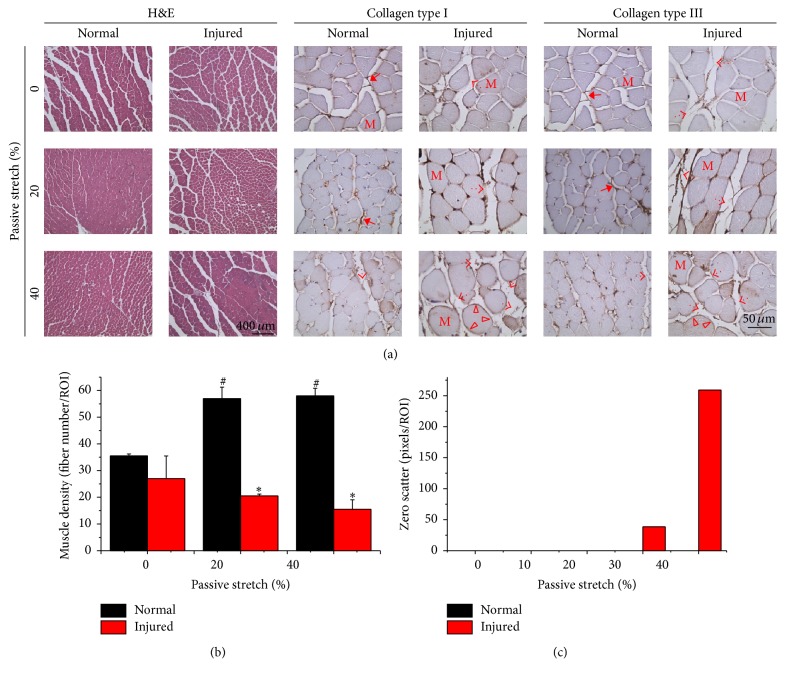
The normal and injured muscles were passively stretched and fixed at 0, 20, and 40% of deformation to show the histological appearance of muscle and collagen fibers (a). Hematoxylin and eosin (H&E) staining revealed morphological and compositional changes (left) among different stains in normal and injured muscles. Immunohistochemistry (IHC) staining of type I (middle) and type III (right) collagen fibers (brown color) showed the rupture of connective collagen fibers (open arrow). The cross-sectional area of the muscle fiber decreased when normal muscle was subjected to passive stretch, whereas increased area was found in the muscle fibers of injured tissue. The quantified results demonstrated significant decreases in muscle density in injured muscles under passive stretch at 20 and 40% strains (b). Increased pixel numbers for zero scatterer within the region of interest (ROI) were also observed in injured muscles when deformation by passive stretch was increased (c). M: muscle fiber; solid arrow: intact collagen fibril; open arrow: ruptured fibril; open triangle: endomysium around the muscle fiber. Bar in H&E image = 400 *μ*m. Bar in IHC image = 50 *μ*m.

The factors possibly related to mechanical stress independence when scanning Nakagami images in injured muscle in cross-sectional images were further evaluated by histology. To investigate how structure changes in muscle during passive stretching, histology was performed after fixing the muscle tissue during different strains (Figure 6(a)). Since Nakagami parameters may indicate scatterer properties and reflect tissue inflammation, collagen fiber arrangement, muscle fiber density, and tissue inflammatory markers were analyzed in both normal and injured muscles. H&E staining demonstrated the cross-sectional features of normal and injured muscle fibers under 0, 20, and 40% passive stretch (Figure 6(a), left part). Collagen is a key structure for determining the mechanical properties in biological tissue; it is also an important ultrasonic scatterer. In muscle tissue, collagen type I and type III are the main components of the perimysium and endomysium. Collagen structures under various levels of passive stretch in normal and injured muscle were observed by IHC staining of collagen type I (Figure 6(a), middle) and type III (Figure 6(a), right). In normal muscle, the arrangement of existing type I and type III collagen fibers was observed around the muscle fibers and fascicles. Collagen was also found to act as a connection between the fibers and fascicles (solid arrows). The connected fibrils existed in normal tissue at 0 and 20% strain but started to tear off after 40% strain was achieved (open arrows). In injured muscles, both the collagen I and III fibers were present in fragments at 0, 20, and 40% strains (open arrows). However, the collagen fibrils surrounding the endomysium were still observed even after 40% strain was achieved in injured muscle (open triangles). In the current study, we did not observe inflammatory responses as indicated by IHC staining of BLT1 and COX2 in injured muscles.

Contrary to the normal muscle, the muscle fibers in injured tissues showed an interesting feature of enlargement in cross-sectional areas when deformation was increased under passive stretch (Figure 6(a)). Similar to the regions chosen as ROIs in ultrasonic images, the muscle density was quantified by the average fiber number from four regions of the histological section. Significant increases in fiber numbers were found in healthy muscle when 20 and 40% passive stretch was applied, whereas decreased fiber numbers were observed in injured tissues (Figure 6(b)). The pixel numbers of zero scatterer also increased significantly when deformation was increased in the injured muscle (Figure 6(c)). Taken together, these results suggest that scanning of ultrasonic images perpendicular to muscle fiber orientation in combination with Nakagami analysis can distinguish muscle injury.

## 4. Discussion

The current study demonstrated that ultrasonographic characteristics can be used in combination with biomechanical factors to analyze the structural integrity of calf muscles following overstretching injuries. Our results indicate that overstretch damage could be detected by ultrasonic parameters, particularly echogenicity and Nakagami, during applied passive strain. In addition, HFU scanning directions are important for observing damage in muscle fibers.

Although many imaging modalities are capable of detecting focal damage, such as tears, mechanical function in muscle tissue is generally not detectable. Here, we observed that echogenicity of ultrasonic images can reflect the loading situation in the muscle tissue. The relationship between mechanical properties and ultrasonic parameters was previously studied in normal tendons [25]. Echogenicity in ultrasonography followed the strain curve in the tendon and showed reductions in damaged tendons [26]. We examined muscle strength (strains) and demonstrated a similar decreasing pattern in damaged muscle compared to normal tissue. Our results showed high correlation coefficients in echogenicity, which reflect the mechanical behavior of healthy muscle, regardless of HFU scanning direction. In damaged muscles, lower echogenicity could also be explained by decreased mechanical stress and we showed a moderate correlation to mechanical stress during passive stretching. The overstretch damage caused nonrecoverable deformation in the biological contractile tissue and resulted in lowering the stress during mechanical testing [27]. During stress relaxation tests in injured tendons, minimal changes in echogenicity were observed to result from decreased stress at the same strain and reduced viscoelastic response [26]. Mechanical behavior decreases in biological contractile tissue could be explained by permanent damage occurring in skeletal muscle tissue. Decreased ultrasonic parameters observed in the original state of the injured muscle for current study may reflect nonrecoverable deformation in the tissue. This indicates that echogenicity could potentially provide information regarding the mechanical state of skeletal muscle tissue.

In skeletal muscle tissue, force is transmitted by muscle fibers as well as by intramuscular connective tissues composed of ECM. During passive stretch testing, muscle fibers only play a minor role and the ECM acts as the stiff structure that participates in stress-strain curves [28, 29]. Within normal range, passive forces rearrange collagen fibers and condense the ECM composition, which contributes to increased stiffness in the entire tissue [6, 30]. The mechanics of ECM has been elucidated, from muscle fiber to fiber bundles, and highlights the mechanical contribution of muscle bundles within ECM [31, 32]. These tissue characteristics are potential physical parameters that affect scattering of Nakagami signals. Tissue stiffness can refer to the scatterer's acoustic properties and tissue density is relative to the concentration of scatterers [33, 34]. To determine which parameters were major factors causing the changes observed through Nakagami, we still have to consider the mechanical behavior and microstructure of collagen apparent in skeletal muscle tissue. In the current study, overstretch caused partial damage of collagen fibers in connective tissues but not in collagen around the endomysium (Figure 6(a)). The remaining endomysium collagen is still wrapped on the muscle fiber and showed its mechanical behavior in response to passive stretching in longitudinal Nakagami images. The ruptured interconnected collagen fibers showed significant decreases in muscle mechanical properties that can be represented by Nakagami images in cross-sectional orientation. These results suggest that the major loss of mechanical behavior can contribute to ruptured collagen fibrils in the overstretched injured muscle tissue. In addition, it is plausible that the interconnected collagen fibers are responsible for ultrasound scattering of cross-sectional Nakagami images in skeletal muscle. Taken together, the cross-sectional Nakagami images provide information regarding structural integrity at different levels of passive stretching and closely correlate to collagen fiber arrangement and histological architecture.

Cross-sectional Nakagami images provide fine structural details when different levels of passive force are applied to the muscle. These details can be closely correlated to histological structures. In the current study, we used the calf muscle due to the simple orientation and spindle architecture. Another factor that has to be considered was density, although previous findings indicate that small changes in muscle volume and contraction have insignificant effects on ultrasonic backscatter in tissues from healthy muscle specimens [7, 35]. However, changes in muscle density within the injured muscle were obvious (Figure 6(b)). High correlations were found between muscle density and Nakagami parameters in cross-sectional images (data not shown). Ultrasonography of cross sections would be better for observing damaged muscle tissue because most skeletal muscles in the body are multiheaded and the angle of muscle fibers is not parallel to the axis of the muscle tendon.

Other factors may also influence backscatter ultrasound signals and result in failure to represent the concentration of scatters in Nakagami images, including blood vessels [18, 36]. In the current study, we used an* ex vivo* system to test the mechanical and ultrasonic properties of injured muscle and we were able to exclude the possibility of vascular remodeling after muscle injury* in vivo*. The method used in this study provides a great improvement to the assessment of musculoskeletal mechanics. However, unlike increased echogenicity detected in fractured bone that reflects inflammation response [17], the current study did not detect increased inflammatory markers in the injured muscle. The* ex vivo* system simplified several consequences of strain injury in muscle: muscle guarding, swelling, and neutrophil/macrophage infiltrations. A system capable of noninvasively measuring skeletal muscle tissue mechanics and quantifying the damaged mechanics* in vivo* could confirm these findings. An ultrasound-based method could potentially, noninvasively, provide information regarding mechanical behavior in muscle tissue, particularly when the results can be compared to a healthy normal tissue in the same subject. Based on the diameter of muscle fiber and tissue properties, we think the cross-sectional Nakagami image has high potential to translate into clinical settings for human application. The common ultrasound systems in orthopedic clinics have the frequencies between 10 and 15 MHz which can provide an image resolution higher than 300 *μ*m in Nakagami image and a scanning depth for around 5 cm. Thus, the Nakagami function can be integrated into the current ultrasound settings to scan most skeletal muscles in our human body. For these superficial muscles that are easy to suffer the strain injury, higher frequency (above 15 MHz) of HFU imaging can be established to reveal the detailed structural changes in muscle tissues. Potential applications for this information include the surgical follow-up, evaluation of rehabilitation and the healing process after strain injury, and sport-related studies.
